# Vitamins A, E and D and cancer risk in BRCA1 carriers

**DOI:** 10.1186/1897-4287-10-S3-A10

**Published:** 2012-04-20

**Authors:** K Kąklewski, D Gackowski, K Durda, T Huzarski, J Gronwald, T Dębniak, A Tołłoczko, O Ashuryk, A Jakubowska

**Affiliations:** 1 Pomeranian Medical University and Read Gene SA, Szczecin, Poland

## 

Vitamins are important in various biochemical and physiological processes and are essential for the normal functioning of our bodies. Several studies suggested the role of vitamins A, E and D in carcinogenesis.

The aim of this study was to analyze an association between concentration of vitamins A, E and D in serum and cancer risk in BRCA1 mutation carriers.

Study was conducted on 99 patients affected by breast cancer and 198 healthy women matched to the cases by year of birth, adnexectomy, smoking and cancer family history. All cases and controls were carriers of Polish BRCA1 founder mutation (5382insC, C61G, 4153delA).

The concentration of vitamins A, E and D was quantitatively measured by HPLC chromatography (Flexar HPLC, Perkin Elmer). The mean levels of analyzed vitamins were compared for cases and controls. For cancer risk assessment, individuals were divided into quartiles, based on the distribution of tested vitamin’s levels.

Neither vitamin D, nor vitamin E were significantly associated with the disease risk. However, we found significant association of vitamin A concentration with cancer risk. Individuals in the quartile with the lowest concentration had the highest risk of breast or ovarian cancer.

**Table 1:** Breast cancer risk in BRCA1 carriers depending on vitamin A concentration

Vit A (μg/l)	Cases (n=99)	Controls (n=194)	OR	p-value
0,60 – 1,32[	35(35,4%)	38(19,6%)	1,000	-
[1,32 – 1,55[	22(22,2%)	51(26,3%)	0,468	0.02190
[1,55 – 1,80[	21(21,2%)	52(26,8%)	0,438	0.15977
[1,80 – 2,55	21(21,2%)	53(27,3%)	0,430	0.01341

